# Conditional Mapping Identified Quantitative Trait Loci for Grain Protein Concentration Expressing Independently of Grain Yield in Canadian Durum Wheat

**DOI:** 10.3389/fpls.2021.642955

**Published:** 2021-03-24

**Authors:** Yuefeng Ruan, Bianyun Yu, Ron E. Knox, Wentao Zhang, Asheesh K. Singh, Richard Cuthbert, Pierre Fobert, Ron DePauw, Samia Berraies, Andrew Sharpe, Bin Xiao Fu, Jatinder Sangha

**Affiliations:** ^1^Swift Current Research and Development Centre, Agriculture and Agri-Food Canada, Swift Current, SK, Canada; ^2^Aquatic and Crop Resource Development, National Research Council Canada, Saskatoon, SK, Canada; ^3^Aquatic and Crop Resource Development, National Research Council Canada, Ottawa, ON, Canada; ^4^Grain Research Laboratory, Canadian Grain Commission, Winnipeg, MB, Canada

**Keywords:** conditional mapping, quantitative trait loci, grain protein concentration, durum wheat, grain yield

## Abstract

Grain protein concentration (GPC) is an important trait in durum cultivar development as a major determinant of the nutritional value of grain and end-use product quality. However, it is challenging to simultaneously select both GPC and grain yield (GY) due to the negative correlation between them. To characterize quantitative trait loci (QTL) for GPC and understand the genetic relationship between GPC and GY in Canadian durum wheat, we performed both traditional and conditional QTL mapping using a doubled haploid (DH) population of 162 lines derived from Pelissier × Strongfield. The population was grown in the field over 5 years and GPC was measured. QTL contributing to GPC were detected on chromosome 1B, 2B, 3A, 5B, 7A, and 7B using traditional mapping. One major QTL on 3A (*QGpc.spa-3A.3*) was consistently detected over 3 years accounting for 9.4–18.1% of the phenotypic variance, with the favorable allele derived from Pelissier. Another major QTL on 7A (*QGpc.spa-7A*) detected in 3 years explained 6.9–14.8% of the phenotypic variance, with the beneficial allele derived from Strongfield. Comparison of the QTL described here with the results previously reported led to the identification of one novel major QTL on 3A (*QGpc.spa-3A.3*) and five novel minor QTL on 1B, 2B and 3A. Four QTL were common between traditional and conditional mapping, with *QGpc.spa-3A.3* and *QGpc.spa-7A* detected in multiple environments. The QTL identified by conditional mapping were independent or partially independent of GY, making them of great importance for development of high GPC and high yielding durum.

## Introduction

Durum wheat [*Triticum turgidum* L. subsp. *durum* (Desf.) Husn.], is an economically important crop owing to its unique characteristics contributing to semolina for the production of pasta products and other traditional foods such as flat breads, couscous and bulgur (Giraldo et al., 2016). Grain protein concentration (GPC) is an important trait in durum wheat cultivar development. It is a major determinant of the nutritional value of grain, rheological properties of the dough for pasta making, and end-use product quality, and thus the economic value of the crop. GPC is a complex quantitative trait controlled by multiple genomic loci that interact with each other (Nigro et al., 2019). Selection for high GPC in durum wheat is also confounded by the interference of environmental effects at varying degrees. Therefore, multiple combinations of genotype and environment are required for screening GPC in breeding programs. Simultaneous selection of GPC and grain yield (GY) is difficult due to a negative correlation often observed between these two traits in most genetic backgrounds and growing environments (Blanco et al., 2002, 2006; Groos et al., 2003; Bogard et al., 2011). However, shifting the negative correlation has been demonstrated by selecting simultaneously for both GPC and GY (DePauw et al., 2007).

Understanding the genetic basis of GPC in cultivars in their target environment is the key to the deployment of marker assisted selection (MAS) in durum breeding programs for the maintenance or improvement of grain quality. Studies conducted to dissect the genetic basis of GPC in durum wheat have identified quantitative trait loci (QTL) on almost all chromosomes as summarized by Kumar et al. (2018). Among the reported QTL, a few showed major effects while many produced minor effects. Also, most of the identified QTL were environmentally dependent and not stable across various environments. A well-known QTL for GPC is *Gpc-B1* on chromosome 6BS. The high GPC allele of this QTL was identified from a wild tetraploid (*Triticum turgidum* L. ssp. *dicoccoides*) accession FA-15-3 originating in Israel (Avivi, 1978). *Gpc-B1* was mapped on chromosome 6BS accounting for 66% of the variance in GPC (Joppa et al., 1997; Olmos et al., 2003). The ancestral wild allele of *GPC-B1* encodes a NAC transcription factor (NAM-B1) and is associated with increased grain protein, Zn, and Fe concentration and also accelerates senescence of flag leaves in wheat (Uauy et al., 2006). Modern wheat varieties carry a non-functional NAM-B1 allele and the functional *Gpc-B1* allele has been introgressed into elite cultivars of durum and bread wheat (Chee et al., 2001; Tabbita et al., 2017; Bokore et al., 2019). However, wild type *GPC-B1* allele has larger negative effects on yield components in durum wheat, in addition to the undesirable effect of increasing semolina ash concentration (Tabbita et al., 2017).

QTL mapping analysis of GPC was often conducted without considering GY and yield components. However, some recent studies, taking into account GY and yield components simultaneously, have led to the identification of GPC loci without negative effects on yield-related traits (Blanco et al., 2002, 2012; Suprayogi et al., 2009; Rapp et al., 2018; Nigro et al., 2019). For example, a few studies identified GPC QTL without negative effects on GY by mapping grain protein deviation (GPD) derived from the regression of GPC and yield in the diverse durum panel (Rapp et al., 2018; Nigro et al., 2019). Such loci are useful for simultaneous genetic improvement of GPC and GY. A statistical procedure proposed by Zhu (1995) was used for analyzing conditional genetic effects for single developmental traits, which applies the same statistical principle as GPD approach (Rapp et al., 2018; Nigro et al., 2019) to analyze correlated traits including grain protein and yield. This conditional analysis is used to estimate the trait values based on no variation in genetically correlated traits, a method that is very similar to the estimation of adjusted values in a covariance analysis eliminating the influence of correlated traits on the genetic effects of the QTL for targeted traits (Zhao et al., 2006). This model has been further developed to analyze the contribution of each component trait to a complex trait and also to dissect the genetic interrelationship between closely related traits. Conditional QTL mapping has been successfully used for evaluating QTL effects on the target traits conditional on their component traits such as grain yield in rice (Guo et al., 2005) and spike extension length on plant height in wheat (Li C. et al., 2020). Genetic relationship between related traits at QTL level were investigated for oil content in rapeseed with respect to protein content (Zhao et al., 2006), popping expansion volume of maize conditional on grain weight per plant and 100-grain weight (Li et al., 2008), GPC conditional on grain starch content in wheat (Deng et al., 2015), and protein content and oil content in soybean (Li X. et al., 2020). In addition, conditional QTL mapping has been used to elucidate environmental effects on QTL expression based on trait values conditioned on different environments (Xu et al., 2014; Fan et al., 2019). Conditional analysis was performed to study the effects of nitrogen (N) and phosphorus (P) fertilization on the expression of QTL for yield and nitrogen-related traits (Xu et al., 2014) and low N-stress induced QTL in wheat (Fan et al., 2019). Furthermore, conditional QTL mapping can identify additional QTL that are undetectable in traditional mapping.

Using conditional mapping, we expected to identify QTL for GPC that are independently expressed from GY, which can facilitate simultaneous selection of high protein concentration and high GY in durum breeding. Therefore, the objectives of this study were to: (1) identify QTL underlying GPC in Canadian durum wheat in particular those that are stable QTL across multiple environments, (2) specify the QTL for GPC without negative correlated effects on GY by conditional QTL analysis.

## Materials and Methods

### Population, Field Trials, and Trait Measurement

A durum population of 162 doubled haploid (DH) lines derived from Pelissier × Strongfield was used in this study. Strongfield is a registered Canada Western Amber Durum variety with strong gluten, high GPC, and low cadmium developed at the Swift Current Research and Development Centre, Swift Current, SK (Clarke et al., 2005). Pelissier, a selection from an Algerian landrace introduced by way of the United States of America, is a founder parent in the Canadian durum wheat gene pool (Clarke et al., 2010). It has high cadmium and lipoxygenase. The DH lines, along with their two parents and controls were tested in field trials at the South Farm of SCRDC (latitude: 50°17′N; longitude: 107°41′W; elevation 825 m) on a Swinton loam (Orthic Brown Chernozem) in four-row plots (2.74 m^2^/plot) as a randomized complete block design with two replicates. Each trial was grown at two seeding dates with a 1-week interval (early, E; late, L) each year from 2014 to 2016, and only the early seeding date in 2017 and 2018. Each seeding date trial was grown at a different plot-land. Plots were harvested into individual bags using a plot combine. The GY of each plot was measured by a weighing balance and expressed in kg ha^−1^. The semolina GPC was measured using Near Infrared (NIR) Spectroscopy (Foss NIR 6500) and expressed as a percentage at a 13.5% moisture basis. For phenotypic data analysis and QTL mapping, each seeding date in each year was considered as one environment providing a total of eight environments labeled as E14, L14, E15, L15, E16, L16, E17 and E18. Pre-plant soil testing was conducted each year to determine the rate of fertilizer application. The fertilizers were applied to target 112 kg ha^−1^ for nitrogen, 70 kg ha^−1^ for phosphorus and 22.4 kg ha^−1^ for sulfur. The soil is naturally high in potassium and did not require additional application.

### Statistical Analysis

The statistical summary and Shapiro-Wilk normality test were conducted in R (R3.3.2, https://www.r-project.org/). Pairwise phenotypic correlations between environments and between traits were calculated using the Pearson correlation coefficient in the R package Hmisc (version 4.2-0, http://cran.r-project.org/web/packages/Hmisc/index.html).

Analysis of variance (ANOVA) and heritability estimate were performed using the PROC MIXED procedure of SAS 9.3 (SAS Institute, Cary, NC, USA) as described by Ruan et al. (2020). In the mixed model, DH lines (genotypes, G) were considered as fixed effects, while environments (E), genotype × environment (G × E) interactions and replications nested in environments were considered as random effects. The heritability of GPC was calculated as the ratio of the genetic variance and the phenotypic variance across environments using $\sigma_{\text{g}}^{2}$/($\sigma_{\text{g}}^{2}$ + $\sigma_{\text{ge}}^{2}$/y + $\sigma_{\text{ε}}^{2}$/yr), where $\sigma_{\text{g}}^{2}$, $\sigma_{\text{ge}}^{2}$, and $\sigma_{\text{ε}}^{2}$ were estimates of genotype (G), genotype × environment (G × E) interaction, and residual variance (error), respectively, and y and r represented the numbers of environment and replication. The heritability of GPC in each environment was calculated by using $\sigma_{\text{g}}^{2}$/($\sigma_{\text{g}}^{2}$ + $\sigma_{\text{ε}}^{2}$/r), where $\sigma_{\text{g}}^{2}$ and $\sigma_{\text{ε}}^{2}$ were estimates of genotype and residual variance, respectively, and r represented the numbers of replication. For the estimations of the heritability, all effects were considered random.

### Genetic Map and QTL Mapping

QTL mapping was performed using the genetic map of Pelissier × Strongfield reported by Ruan et al. (2020). The Infinium iSelect Wheat 90K SNP chip was used for genotyping. A total of 1,212 polymorphic SNP markers with <30% missing data were used for genetic map construction, which lead to the identification of 25 linkage groups (LGs). LGs were assigned to chromosomes based on comparison with an existing high-density SNP-based consensus map of durum wheat (Maccaferri et al., 2019). Mean values of GPC from two replicates in each environment were used for the detection of QTL. Outliers of trait values were detected and removed using a Z-score transformation with a threshold of 3. QTL detection was performed using composite interval mapping (CIM) in WinQTL Cartographer v.2.5 software (Wang S. et al., 2012) (http://statgen.ncsu.edu/qtlcart/WQTLCart.htm). The same parameters as described by Ruan et al. (2020) were used for CIM. QTL detected in different environments were considered the same if the confidence intervals (CI) overlapped and the additive effect was contributed by the same parent. QTL mapped in at least one environment explaining more than 20% of the phenotypic variance or mapped in at least two environments with PVE ≥ 10% were considered as major QTL (Raihan et al., 2016; Zhao et al., 2016). The QTL detected in two or more environments are considered as stable QTL. Graphical representation of linkage groups and QTL on genetic map was performed using MapChart 2.2 software (Voorrips, 2002). Haplotypes were assigned using R package Haplotyper.

The conditional GPC values [GPC|GY, GPC conditional on grain yield (GY)] from each environment were calculated using QGA Station 2.0 (Zhu, 1995) (http://ibi.zju.edu.cn/software/qga/v2.0/index.htm). The conditional phenotypic values (GPC|GY) are the net trait values of GPC independent of variation in GY. QTL mapping for conditional GPC values was performed using the same method as above for the traditional QTL mapping. The QTL identified were defined as conditional QTL. When the QTL identified by the two methods (traditional and conditional) had overlapping CIs, they were assumed to be identical. All reported QTL were designated according to the Recommended Rules for Gene Symbolization in Wheat (http://wheat.pw.usda.gov/ggpages/wgc/98).

Best linear unbiased prediction (BLUP) is a popular method used for analyzing multi-environment trials (Xiao et al., 2016; Choudhury et al., 2019). To eliminate the influence of environmental effects on phenotypic variation, BLUP value of GPC for each line across all environments was estimated using the linear model in R package lme4 (Bates et al., 2015). The BLUP values of DH lines were used as trait data for QTL mapping across all environments as described by Xiao et al. (2016).

### Comparison With Previously Reported QTL

Sequences of the 90K SNPs were downloaded from the Kansas University SNP marker database (http://wheatgenomics.plantpath.ksu.edu/snp/). Sequences of SSR markers were retrieved from the GrainGenes database (https://wheat.pw.usda.gov/GG3/). Sequences of DArT markers were downloaded from DArT P/L website (https://www.diversityarrays.com/technology-and-resources/sequences). Physical map positions of SNP, SSR and DArT markers on the genome of durum wheat cv. Svevo (Maccaferri et al., 2019) were aligned using BLASTn at the Svevo portal (https://d-data.interomics.eu). QTL reported in the literature and identified in this study were projected onto the genome of durum cv. Svevo by projecting a single marker closest to the QTL peak position. QTL markers on the physical map of Svevo were drawn using PhenoGram software (http://visualization.ritchielab.org/phenograms/plot).

## Results

### GPC Variation in DH Population Across Multiple Environments

**Figure 1:**
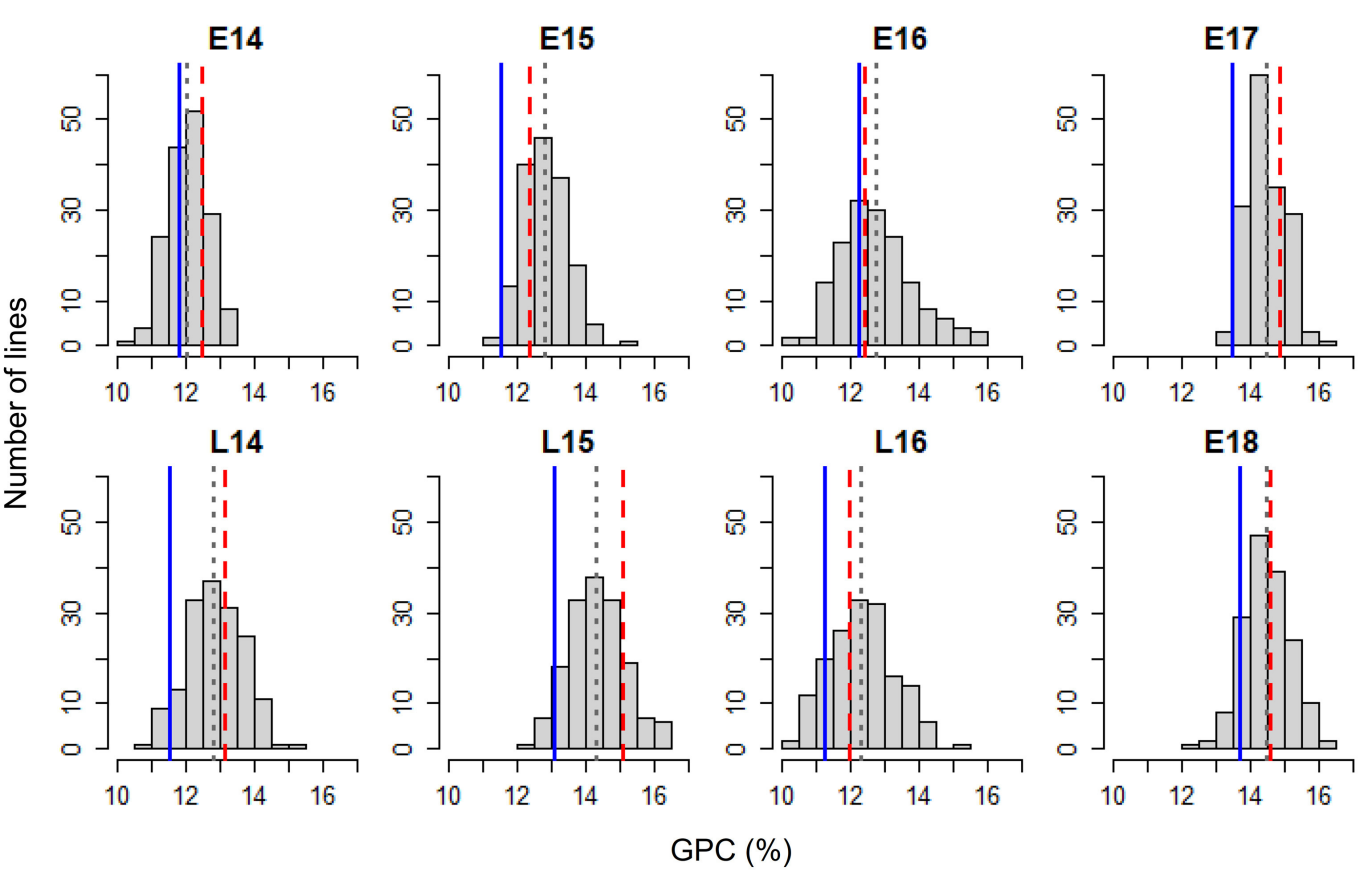
Frequency distribution of grain protein concentration (GPC) in the Pelissier × Strongfield population from 2014 to 2018 field trials with two seeding dates in each year (early, E; late, L) during 2014–2016 and only early seeding date in 2017–2018. Blue solid lines represent Pelissier; red dashed lines represent Strongfield; gray dotted lines indicate mean values of the population.

**Figure 2:**
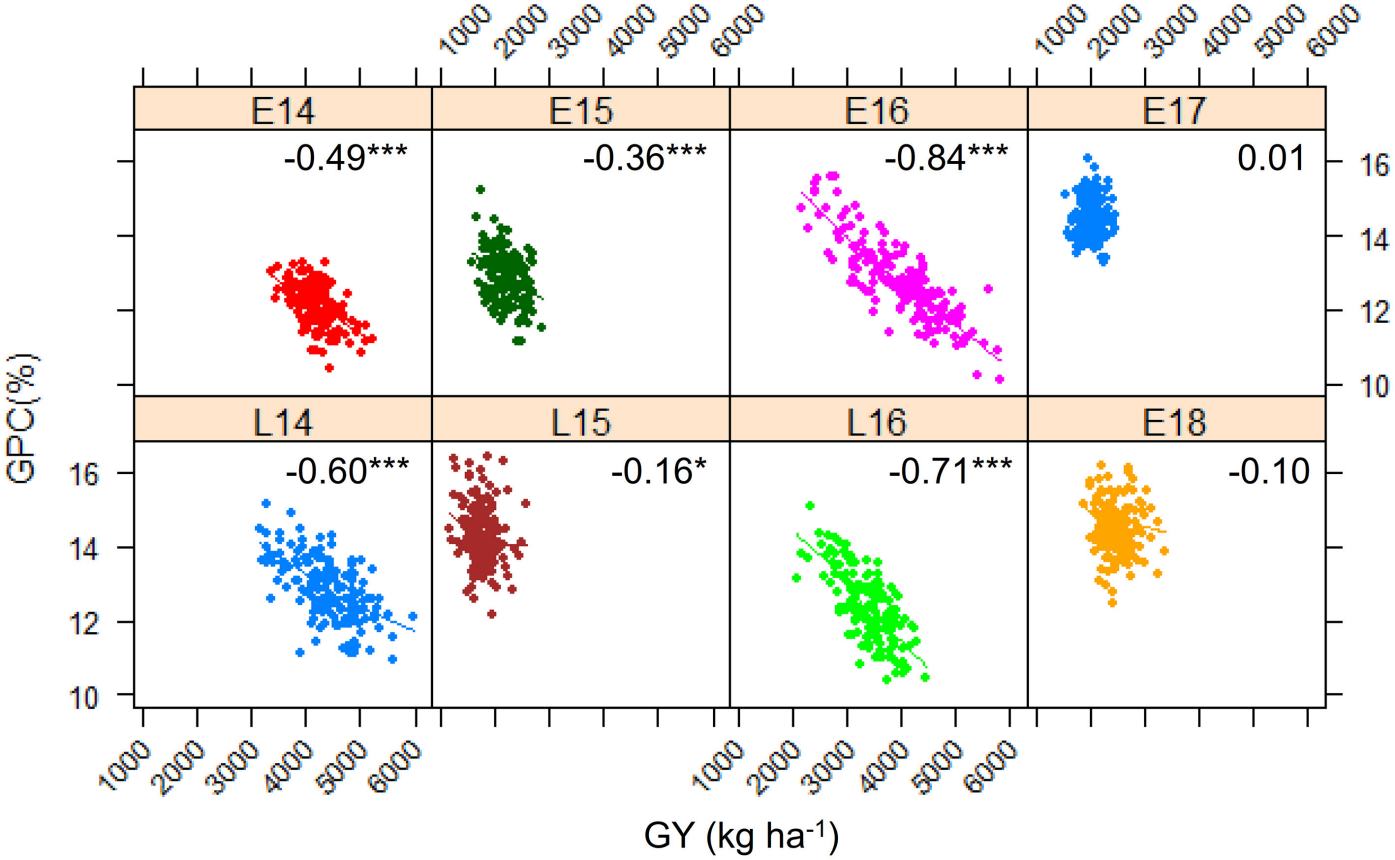
Scatterplot and Pearson correlation coefficient between grain protein concentration (GPC) and grain yield (GY) of the Pelissier × Strongfield population in eight environments. Symbol *indicates significance *p* < 0.05 and ***p < 0.001.

Figure 1 shows the frequency distribution of GPC in the DH population derived from Pelissier × Strongfield across eight environments from 2014 to 2018. Summary statistics including mean values and standard deviation (SD) of the population mean, minimum and maximum values, range and the probability associated with Student's *t*-test for the parental means in each environment is shown in Table 1. The distribution of GPC was normal across all environments except environment E16 as indicated by the *p*-value of the Shapiro-Wilk normality test. The parental line, Strongfield, had significantly higher GPC than Pelissier across all environments except in E16 (Table 1). The individual DH lines had extreme GPC values in all environments and displayed bi-directional transgressive segregation for GPC, as shown by the maximum and minimum values relative to the parents. The mean GPC of the DH population was closer to the parent Strongfield than to the parent Pelissier in most environments. The GPC of the population had the highest mean value in environment E18 (mean = 14.5%) and the lowest mean value in E14 (mean = 12.1%). The largest GPC range was observed in environment E16 and smallest was observed in E17. Moderate Pearson correlation coefficients (0.3–0.67) were observed among DH lines across environments (Supplementary Figure 1). Significant negative correlations from −0.16 to −0.84 were observed between GPC and GY in multiple environments except for E17 and E18 (Figure 2). In general, the higher the grain yield, the stronger the negative correlation between GPC and GY.

**Table 1:** Mean, standard deviation, minimum and maximum, coefficient of variation, probability associated with the Shapiro-Wilk normality test of grain protein concentration (GPC) across environments for the Pelissier × Strongfield population, heritability in each environment, GPC mean of the parents and the *p-*value of Student's *t-*test for significance between the two parents.

	**DH lines (population)**					**Parents**		
**Env**	**Mean ± SD (%)**	**Min-Max (%)**	**CV (%)**	***p*-value^a^**	**Heritability**	**Strongfield (%)**	**Pelissier (%)**	***p*-value^b^**
E14	12.07 ± 0.57	10.41–13.3	4.72	0.696	0.49	12.52	11.84	0.044
L14	12.84 ± 0.82	10.96–15.16	6.39	0.741	0.63	13.17	11.55	0.031
E15	12.81 ± 0.67	11.15–15.24	5.23	0.250	0.74	12.34	11.52	0.004
L15	14.33 ± 0.81	12.13–16.43	5.65	0.650	0.75	15.07	13.10	0.004
E16	12.76 ± 1.07	10.09–15.6	8.39	0.010	0.84	12.45	12.27	0.432
L16	12.34 ± 0.95	10.41–15.11	7.70	0.335	0.85	12.03	11.30	0.022
E17	14.47 ± 0.54	13.28–16.06	3.73	0.054	0.64	14.87	13.46	2.7E−05
E18	14.49 ± 0.69	12.48–16.21	4.76	0.921	0.73	14.60	13.71	0.015

a *p-value of Shapiro-Wilk normality test*.

b *p-value of Student's t-test*.

*Env, Environment; SD, standard deviation; Min, Minimum; Max, Maximum; CV, coefficient of variation; DH, doubled haploid*.

### Traditional QTL Mapping Using Phenotypic Data From Single Environments and BLUP Values Across Environments

**Figure 3:**
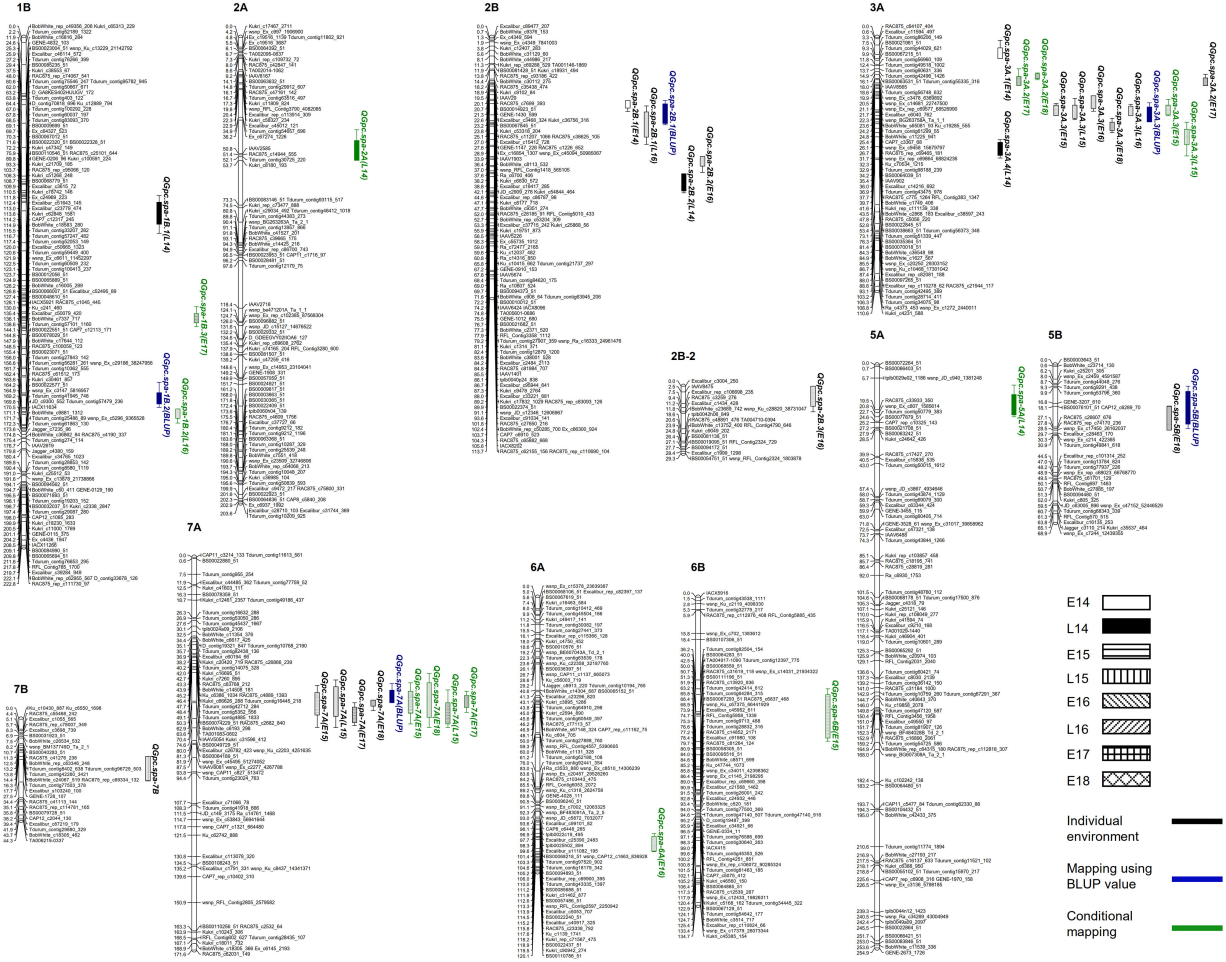
Distribution of quantitative trait loci (QTL) for grain protein concentration (GPC) detected in each environment, by using best linear unbiased prediction (BLUP) values and conditional mapping.

Although a high broad sense heritability of 0.87 was observed for GPC, genotype × environment interactions was significant as revealed by ANOVA (Table 2). Therefore, QTL analysis was first performed for GPC in each environment. Two to three QTL were detected in each environment (Table 3 and Figure 3). A total of 11 QTL were detected across eight environments, seven of which were specific for a single environment. Both parental lines contributed favorable alleles to different QTL (three by Strongfield and eight by Pelissier). Two QTL were detected in at least four environments with slight shifts in the peak position. The most stable QTL, *QGpc.spa-3A.3*, located on chromosome 3A, was detected in five environments with a LOD score range of 5.5–10.7 and explaining 9.4–18.1% of the phenotypic variance (*R*^2^) in each individual environment. Pelissier contributed the higher GPC allele to *QGpc.spa-3A.3*. Another stable QTL, *QGpc.spa-7A*, was detected on chromosome 7A across four environments, explaining up to 14.8% of the phenotypic variance, with the higher GPC allele derived from Strongfield. Two QTL on chromosome 2B, *QGpc.spa-2B.1* and *QGpc.spa-2B.2*, were detected in two out of eight environments. *QGpc.spa-2B.1* explained 15–16% of the phenotypic variance and *QGpc.spa-2B.2* explained 9–13% of the phenotypic variance, with higher GPC allele from Pelissier at both QTL. In addition, seven QTL, on 1B (*QGpc.spa-1B.1*), 2B (*QGpc.spa-2B.3*), 3A (*QGpc.spa-3A.1, QGpc.spa-3A.2* and *QGpc.spa-3A.4*), 5B (*QGpc.spa-5B*), and 7B (*QGpc.spa-7B*), were detected in a single environment with *R*^2^ values ranging from 4.9 to 6.9%. Pelissier and Strongfield contributed trait increasing alleles to five and two QTL detected in a single environment.

**Table 2:** Analysis of variance (ANOVA) of grain protein concentration (GPC) across environments.

**Sources of variation**	**DF**	**Mean squares**
Environment (E)	7	0.9554^*^
Replication/Environment	8	0.0983^*^
DH lines (G)	161	2.3725^****^
G × E Interaction	1,127	0.1799^****^
Error	1,288	0.3130^****^

* p < 0.05;

**** *p < 0.0001; DF, degree of freedom*.

**Table 3:** Quantitative trait loci (QTL) identified for grain protein concentration (GPC) in the Pelissier × Strongfield population in each environment using GPC values and conditional mapping, and using best linear unbiased prediction (BLUP) values across eight environments, the marker at the peak LOD, peak LOD value, the additive effect, *R*^*2*^, and interval in which the LOD score dropped by 2 points from the peak LOD value.

**Chr**	**QTL**	**Env**	**Peak marker name**	**Peak marker ID**	**Peak position (cM)**	**LOD**	**Additive^a^**	***R^**2**^* (%)^b^**	**Interval (2 LOD drop) (cM)**	**Interval makers (2 LOD drop)**	**Interval makers ID (2 LOD drop)**
**QTL mapping with GPC values**											
1B	*QGpc.spa-1B.1*	L14	BS00110546_51	IWB12562	75.9	3.5	0.20	5.7	69.1–85.7	BS00009699_51–GENE-0206_96	IWB6100–IWB31738
2B	*QGpc.spa-2B.1*	E14	Ex_c16854_1307	IWB19970	28.9	7.6	−0.23	16.0	27.6–32.4	RAC875_c1226_652–IAAV1903	IWB53512–IWB34469
		L16	Excalibur_c18417_285	IWB23131	38.8	8.5	−0.37	15.0	30–46.8	wsnp_Ex_c45094_50985067–Kukri_c8177_718	IWA3924–IWB47895
2B	*QGpc.spa-2B.2*	L14	Ku_c10415_662	IWB38099	65.8	5.4	−0.25	9.0	59.6–67.9	Ku_c12037_482–IAAV5674	IWB38293–IWB35071
		E16	Kukri_c25868_56	IWB43196	54.3	8.0	−0.39	13.0	52–59	RAC875_c28185_91–Ra_c72477_2165	IWB56173–IWB52584
2B	*QGpc.spa-2B.3*	E16	IAAV8475	IWB35482	2.5	3.3	−0.25	4.9	0–11.2	Excalibur_c3004_250–Excalibur_c1434_428	IWB24927–IWB22415
3A	*QGpc.spa-3A.1*	E14	BS00021981_51	IWB6837	7.5	4.5	−0.17	9.1	1.3–9.3	Excalibur_c11594_497–Tdurum_contig86206_149	IWB21927–IWB73711
3A	*QGpc.spa-3A.2*	E17	wsnp_Ex_c14681_22747500	IWA1922	20.5	4.7	−0.17	9.8	16.1–21.1	BS00063531_51–wsnp_Ex_rep_c69577_68526990	IWB9076–IWA5617
3A	*QGpc.spa-3A.3*	E15	Ku_c70534_1215	IWB39901	32.3	6.8	−0.23	12.8	28.9–34.2	wsnp_Ex_rep_c69864_68824236–BS00064039_51	IWA5650–IWB9177
		L15	Tdurum_contig98188_239	IWB74032	32.9	9.5	−0.35	18.1	26.7–36	RAC875_rep_c69465_181–Excalibur_c14216_692	IWB62575–IWB22387
		E16	RAC875_rep_c69465_181	IWB62575	27.7	10.7	−0.46	18.1	25.5–32.3	CAP7_c3367_68–Ku_c70534_1215	IWB14015–IWB39901
		L16	Ku_c70534_1215	IWB39901	32.3	5.5	−0.30	9.4	30.1–34.2	wsnp_Ex_rep_c69864_68824236–BS00064039_51	IWA5650–IWB9177
		E18	RAC875_c775_1264	IWB60468	37.7	8.8	−0.28	16.0	35.4–41.6	IAAV902–Kukri_rep_c111139_338	IWB35578–IWB49486
3A	*QGpc.spa-3A.4*	L14	RAC875_c5056_220	IWB58656	47.8	11.7	−0.38	21.5	44.7–52.8	BobWhite_c2868_183–BS00022845_51	IWB2226–IWB7288
5B	*QGpc.spa-5B*	E18	RAC875_c26607_676	IWB55955	24.1	3.5	−0.18	6.6	18–28.5	BS00076101_51–wsnp_Ex_c17450_26162037	IWB10851–IWA2220
7A	*QGpc.spa-7A*	E15	TA001083-0602	IWB65337	63.6	5.4	0.21	9.8	56–74.4	RAC875_c2682_840–BS00049729_51	IWB55990–IWB8555
		L15	BobWhite_c6193_298	IWB4104	62.9	3.9	0.22	6.9	53.6–74.5	RAC875_c2682_840–BS00049729_51	IWB55990–IWB8555
		E17	IAAV5054	IWB34967	70.4	5.2	0.24	10.8	63.6–73.5	TA001083-0602–BS00049729_51	IWB65337–IWB8555
		E18	BobWhite_c6193_298	IWB4104	62.9	8.3	0.32	14.8	62.4–67	BobWhite_c6193_298–IAAV5054	IWB4104–IWB34967
7B	*QGpc.spa-7B*	L15	GENE-1728_107	IWB32614	21.7	3.6	0.22	6.9	16.3–27.1	Tdurum_contig77503_738–GENE-1728_107	IWB73419–IWB32614
**QTL mapping with BLUP values**											
1B	*QGpc.spa-1B.2*		Tdurum_contig56281_261	IWB72499	156.6	3.4	0.11	4.6	155.3–161.7	BS00023071_51–Tdurum_contig10362_555	IWB7410–IWB66483
2B	*QGpc.spa-2B.1*		IAAV1903	IWB34469	34.9	6.2	−0.15	9.0	27.6–38.2	GENE-1147_226–Kukri_c6830_572	IWB32258–IWB47454
3A	*QGpc.spa-3A.3*		Ku_c70534_1215	IWB39901	32.3	13.4	−0.23	20.7	28.6–36.9	RAC875_rep_c69465_181–Tdurum_contig43475_978	IWB62575–IWB71425
5B	*QGpc.spa-5B*		BS00076101_51	IWB10851	19.1	3.5	−0.11	5.0	9.3–27.8	Tdurum_contig9291_438–RAC875_rep_c74170_236	IWB73824–IWB63010
7A	*QGpc.spa-7A*		BobWhite_c6193_298	IWB4104	62.9	4.5	0.12	6.2	55.3–63.6	BS00074229_51–TA001083-0602	IWB10718–IWB65337
**Conditional QTL mapping**											
1B	*QGpc.spa-1B.2*	L16	Kukri_c30461_857	IWB43857	163.61	4.05	0.20	7.7	162.4–168.6	BS00023071_51–Tdurum_contig10362_555	IWB7410–IWB66483
1B	*QGpc.spa-1B.3*	E17	Tdurum_contig60509_232	IWB72738	122.51	5.12	−0.20	10.2	117.7–126.5	Tdurum_contig52053_149–BobWhite_c16005_289	IWB72238–IWB859
2A	*QGpc.spa-2A*	L14	IAAV2585	IWB34575	50.81	3.52	0.18	7.4	40.4–53.7	Ex_c67274_1226–Kukri_c8180_193	IWB21111–IWB47898
3A	*QGpc.spa-3A.2*	E17	wsnp_Ex_c3478_6369892	IWA3498	19.91	4.70	−0.18	9.3	13.7–21.1	Tdurum_contig60631_336–wsnp_Ex_rep_c69577_68526990	IWB72751–IWA5617
		E18	BS00063531_51	IWB9076	16.11	5.08	−0.23	9.7	15.6–18.6	Tdurum_contig42496_1426–Tdurum_contig56748_632	IWB71206–IWB72529
3A	*QGpc.spa-3A.3*	E15	Ku_c70534_1215	IWB39901	32.31	5.51	−0.21	10.5	27.1–34.2	RAC875_rep_c69465_181–BS00064039_51	IWB62575–IWB9177
		L15	BobWhite_c2868_183	IWB2226	43.51	4.71	−0.27	8.6	37.1–51.8	Tdurum_contig43475_978–BS00022845_51	IWB71425–IWB7288
5A	*QGpc.spa-5A*	L14	wsnp_Ex_c807_1585614	IWA4765	20.81	3.77	0.19	8.0	8.8–22.7	wsnp_JD_c940_1381248–Tdurum_contig50779_383	IWA6226–IWB72119
6A	*QGpc.spa-6A*	E16	Excalibur_rep_c69900_395	IWB31095	107.01	3.62	0.17	7.9	103.3–111.2	Tdurum_contig97520_902–BS00085688_51	IWB74002–IWB11419
6B	*QGpc.spa-6B*	E15	RAC875_c13920_836	IWB53808	52.01	4.40	0.18	8.1	40–63	BS00064283_51–Tdurum_contig42414_612	IWB9241–IWB71115
7A	*QGpc.spa-7A*	E15	TA001083-0602	IWB65337	63.61	4.59	0.19	8.5	54.9–72.9	BS00074229_51–BS00049729_51	IWB10718–IWB8555
		L15	TA001083-0602	IWB65337	68.61	3.22	0.20	5.7	58–74.4	BS00074229_51–BS00049729_51	IWB10718–IWB8555
		E17	IAAV5054	IWB34967	62.91	3.98	0.16	8.3	58.6–67.8	BobWhite_c6193_298–BS00049729_51	IWB4104–IWB8555
		E18	BobWhite_c6193_298	IWB4104	63.61	6.89	0.27	13.3	50.8–74.4	BobWhite_c6193_298–IAAV5054	IWB4104–IWB34967

*Chr, chromsome; Env, environment*.

a *Additive effect; the positive values indicate that the alleles from Strongfield have the effect of increasing the trait value*.

b *R^2^ is the percentage of phenotypic variance explained by each QTL*.

When QTL mapping was conducted using BLUP values across all environments, a total of five QTL were detected on chromosomes 1B, 2B, 3A, 5B and 7A. The phenotypic variance explained by each QTL ranged from 4.6 to 20.7% (Table 3). It is noteworthy that four (*QGpc.spa-2B.1, QGpc.spa-3A.3, QGpc.spa-5B*, and *QGpc.spa-7A*) out of five QTL were also detected by QTL mapping in individual environments. The QTL on 1B, 2B, 3A, and 7B that mapped only in one environment using GPC values were not detected using BLUP values. One additional QTL, *QGpc.spa-1B.2*, was mapped on chromosome 1B using BLUP values with the trait increasing allele attributed to Strongfield.

### Conditional QTL Analysis

Nine QTL were detected on seven chromosomes using conditional QTL mapping (Table 3). These QTL explained 5.7–13.3% of the phenotypic variance with LOD values of 3.2–6.9. Six out of nine QTL had trait increasing alleles from Strongfield, while the other three QTL had favorable alleles from Pelissier. Among the conditional QTL identified, four (*QGpc.spa-1B.2, QGpc.spa-3A.2, QGpc.spa-3A.3*, and *QGpc.spa-7A*) were also detected by traditional QTL mapping. Particularly important, *QGpc.spa-7A* was detected in the same four environments by both conditional and traditional QTL mapping. *QGpc.spa-3A.2* was detected in two environments (E17 and E18) while it was detected only in E17 by traditional mapping. In contrast, *QGpc.spa-3A.3* was detected in two environments (E15 and L15) yet in these two, plus three additional, environments by traditional mapping. QTL detected on chromosome 2B, 5B and 7B by traditional analysis were not identified by conditional mapping. However, five additional QTL on chromosomes 1B (*QGpc.spa-1B.3*), 2A (*QGpc.spa-2A*), 5A (*QGpc.spa-5A*), 6A (*QGpc.spa-6A*), and 6B (*QGpc.spa-6B*) were only identified using the conditional mapping.

### Haplotype Analysis Across Multiple QTL

**Figure 4:**
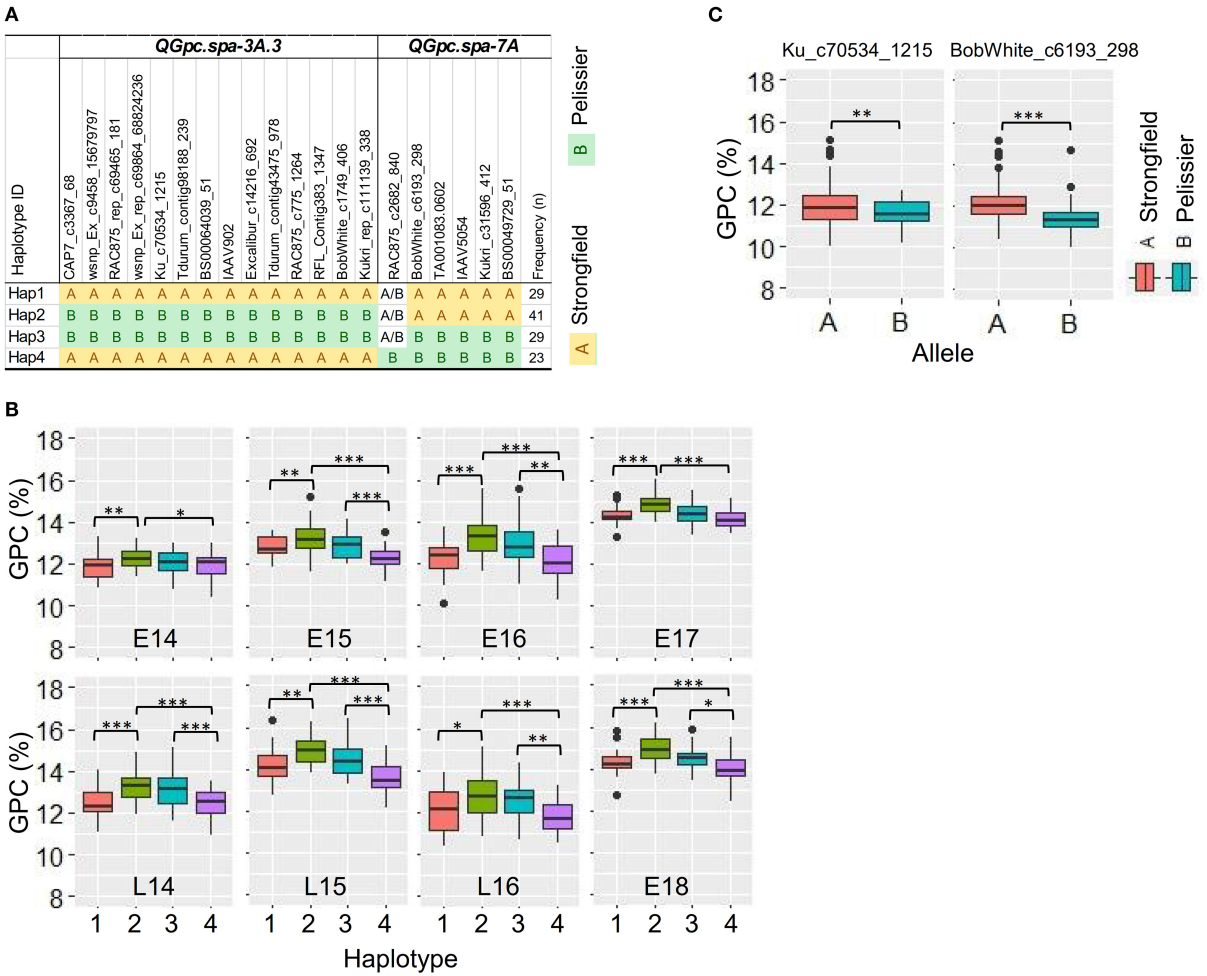
Haplotype analysis of two quantitative trait loci (QTL) in a 2 LOD interval which were detected using best linear unbiased prediction (BLUP) values and identified in two or more environments using traditional and conditional mapping. **(A)** Haplotype block based on SNP markers in each QTL region. **(B)** Boxplots of the phenotypic values corresponding to four different haplotype groups in each environment. Haplotypes containing <3 DH lines were omitted from the table. The DH lines with undetermined haplotype were not shown. **(C)** Boxplots of GPC values in two groups of elite durum lines (*n* = 131) separated on the genotype of the peak marker Ku_c70534_1215 in QTL *QGpc.spa-3A.3* and the peak marker BobWhite_c6193_298 in QTL *QGpc.spa-7A*. GPC, grain protein concentration. *p < 0.05; **p < 0.01; ***p < 0.001 of *t-*test.

To investigate the accumulated effects across multiple QTL of the favorable alleles on GPC, the combined haplotype analysis performed on QTL detected using BLUP values and identified in two or more environments using traditional and conditional mapping was restricted to *QGpc.spa-3A.3* and *QGpc.spa-7A*. The SNPs in the 2 LOD interval of each QTL were used for haplotype analysis. Four different haplotypes (Hap1 to Hap4) were identified at different frequencies, with each haplotype represented in 23–41 DH lines (Figure 4A). The DH lines with Hap2 had the best combination of all favorable alleles at each QTL, as evidenced by the highest GPC across all environments although there was no significant difference between Hap 2 and Hap3 in E14, L14, E16, and L16. The lines with Hap4 had the least favorable combination of the alleles. Significant differences were observed for GPC in the lines with these two haplotype groups Hap2 and Hap4 across all environments. Significant differences between the lines with Hap1 and Hap4 for GPC were also observed across all environments. Except in E14 and E17, GPC was significantly different between the lines carrying Hap3 and Hap4 (Figure 4B). We did preliminary assessment of the effectiveness for MAS using the peak marker *Ku_c70534_1215* (*IWB39901*) in QTL *QGpc.spa-3A.3* and the peak marker *BobWhite_c6193_298* (*IWB4104*) in QTL *QGpc.spa-7A* in a total of 131 elite durum lines that have been phenotyped for GPC and genotyped with the same SNP chip as used for the DH lines in this study. Based on the genotypes of the peak marker in the QTL region, the elite lines were separated into two groups with significantly different GPC means (*t-*test, *p* < 0.01) (Figure 4C). Negative correlation between GPC and GY in different haplotype groups was indicated by the regression lines (Supplementary Figure 2). The regression line of GPC on GY from Hap4 had the largest slope, intercept and *R*^2^, while the regression line of Hap3, carrying favorable allele derived from Pelissier at *QGpc.spa-3A.3*, showed the smallest slope, intercept and *R*^2^. The regression line of Hap 1, carrying favorable allele contributed by Strongfield at *QGpc.spa-7A*, had reduced slope, intercept and *R*^2^ compared with the regression line of Hap4.

### Projection of QTL Onto Reference Genome of Durum Wheat cv. Svevo

**Figure 5:**
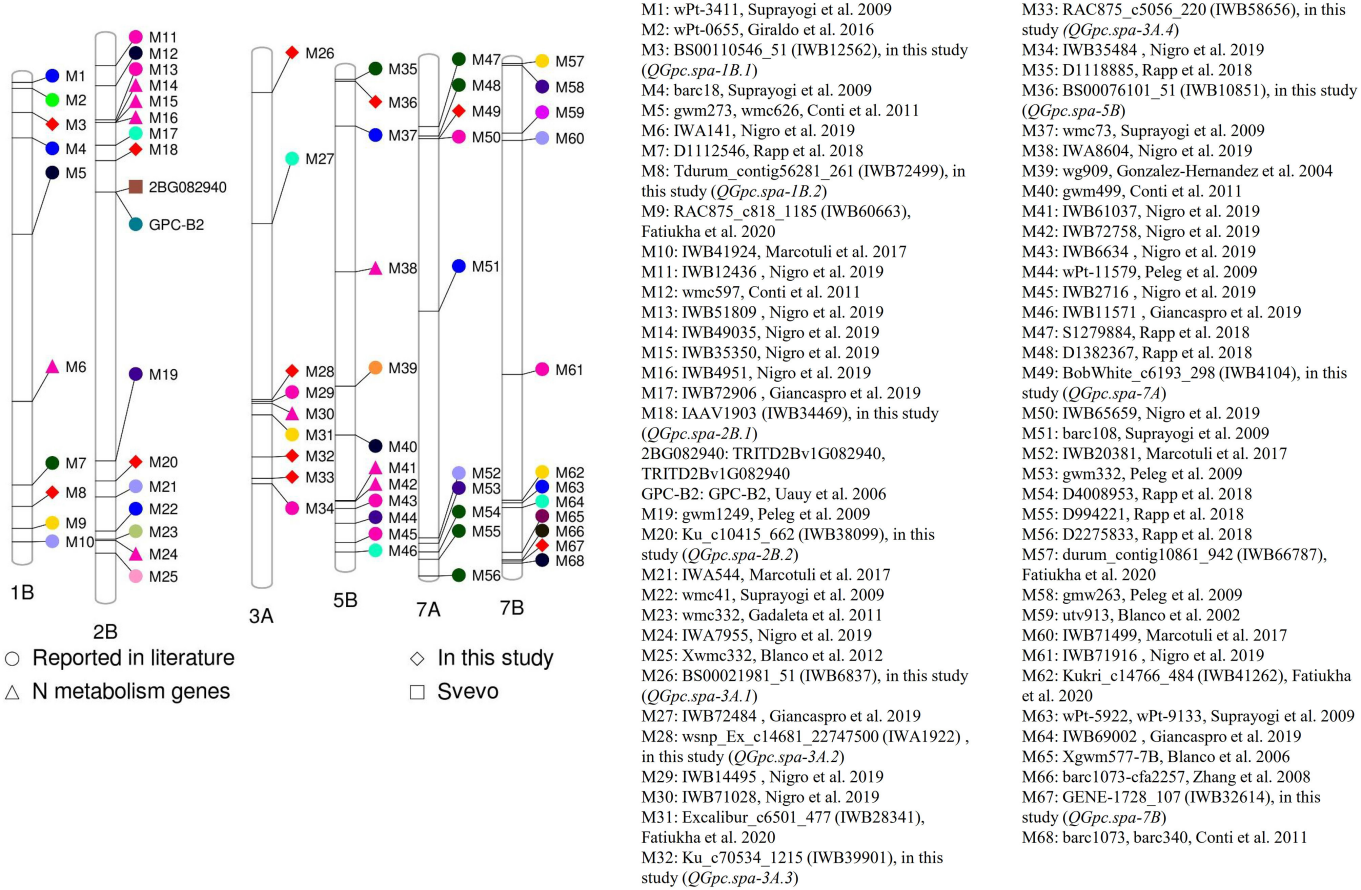
Projection of quantitative trait loci (QTL) for grain protein concentration (GPC) reported in the literature and the QTL identified in two or more environments using traditional and conditional mapping, and using best linear unbiased prediction (BLUP) values in this study onto the reference genome of durum wheat cv. Svevo.

When we projected QTL for GPC identified in this study and those reported in the literature onto the reference genome of durum wheat cv. Svevo, we were able to compare the proximity of each (Supplementary Table 1 and Figure 5). The QTL *QGpc.spa-1B.1* (M3, *IWB12562*) was projected on the short arm of chromosome 1B of durum wheat physical map, ~34.4 Mb away from the QTL (M2, *wPt-0655*) reported by Giraldo et al. (2016) and 35.3 Mb from SSR marker *barc18* (M4) associated with GPC reported by Suprayogi et al. (2009). QTL *QGpc.spa-1B.2* (M8, *IWB72499*) on the long arm of chromosome 1B is 29.7 Mb away from the QTL (M7, *D1112546*) identified by Rapp et al. (2018) and 30.9 Mb from the QTL (M9, *IWB60663*) reported by Fatiukha et al. (2020) in durum wheat.

In the present study, *QGpc.spa-2B.1* was identified on the short arm while *QGpc.spa-2B.2* on the long arm of chromosome 2B. Among the QTL reported by previous studies, the closest QTL to *QGpc.spa-2B.1* is the one (M18, *IWB72906*) with a distance of 22 Mb reported by Giancaspro et al. (2019). *QGpc.spa-2B.2* (M20, *IWB38099*) is 27.7 Mb from the QTL (M19, *gwm1249*) reported by Peleg et al. (2009) and 22.6 Mb from the QTL (M21, *IWA544*) reported by Marcotuli et al. (2017). On the short arm of 3A, Giancaspro et al. (2019) reported a QTL (M27, *IWB72484*) associated with GPC that is 187.6 Mb apart from peak marker of *QGpc.spa-3A.1* (M26, *IWB6837*) detected in this study. The QTL *QGpc.spa-3A.3* (M32, *IWB39901*) on the long arm of chromosome 3A detected in this study and the QTL (M31, *IWB28341*) reported by Fatiukha et al. (2020) are separated by a physical distance of 59.5 Mb. *QGpc.spa-3A.4* (M33, *IWB58656*) is ~7.8 Mb away from SNP (M34, *IWB35484*) reported to be associated with GPC in durum wheat by Nigro et al. (2019). *QGpc.spa-5B* (M36, *IWB10851*) (M35, *D1118885*) on 5B is in close proximity of about 3.15 Mb to the QTL reported by Rapp et al. (2018). *QGpc.spa-7A* (M49, *IWB4104*) on chromosome 7A is in a distance of 2.24 Mb to the QTL (M48, *D1382367*) reported by Rapp et al. (2018) and 2.94 Mb to the QTL (M50, *IWB65659*) reported by Nigro et al. (2019).

## Discussion

### Stable QTL

In the present study, high broad sense heritability was observed for GPC which is similar to the previously published study (Conti et al., 2011), indicating GPC is mainly controlled by genetic factors. The moderate correlations observed among various environments again suggested a substantial genetic component to the variation of GPC. The 11 QTL detected for GPC using traditional mapping indicates the complex quantitative inheritance of many small-to-medium effect QTL. The four QTL, *QGpc.spa-2B.1, QGpc.spa-2B.2, QGpc.spa-3A.3*, and *QGpc.spa-7A*, were repeatedly detected in two or more environments although additive effects of these QTL diverged in the magnitude among different environments. Similarly, QTL × environment interaction was reported for GPC in durum wheat (Conti et al., 2011). The fact that two of these QTL, *QGpc.spa-3A.3* and *QGpc.spa-7A*, were also mapped by using BLUP values suggests they expressed stably across environments. Given that genotype × environment interaction has great effect on durum wheat GPC, the QTL that expressed stably across environments should be valuable in maintaining GPC under selection for germplasm enhancement.

### Comparison With Previous Studies

The physical distance of *QGpc.spa-1B.1* (M3, *IWB12562*) of ~34.4 Mb from the QTL peak marker, *wPt-0655* (M2), reported by Giraldo et al. (2016) and 35.3 Mb from SSR marker, *barc18* (M4), reported by Suprayogi et al. (2009) would suggest they are different loci. But due to the poor resolution of mapping, the possibility that the same gene is functioning in all studies cannot be ruled out. The proximity of QTL *QGpc.spa-1B.2* (M8, *IWB72499*) 29.7 Mb away from the QTL (M7, *D1112546*) identified by Rapp et al. (2018) and 30.9 Mb from the QTL (M9, *IWB60663*) reported by Fatiukha et al. (2020) would again suggest different controlling loci. The physical mapping does suggest *QGpc.spa-1B.1* is different from *QGpc.spa-1B.2*. *TRITD2Bv1G082940*, likely encoding NAM-B2—a paralogous copy of NAM-B1, resides in the interval of *QGpc.spa-2B.1* (156,604,237–231,697,558 bp). *TRITD2Bv1G082940* has four splicing transcripts and encodes the protein with 95.3–99.2% identity with NAM-B2 from *Triticum turgidum* L. subsp. *durum* (Desf.) Husn. (GenBank ABI94355.1) (Supplementary Figure 3).

The 187.6 Mb physical distance between *QGpc.spa-3A.1* (M26, *IWB6837*) and the QTL reported by Giancaspro et al. (2019) (M27, *IWB72484*) indicates these two QTL are different. Because *QGpc.spa-3A.2* is only 3.21 Mb from the QTL peak marker, *IWB14495* (M29), reported by Nigro et al. (2019), they are most likely the same QTL. Nitrogen metabolism related SNP *IWB71028* (M30) (Nigro et al., 2019) is at a distance of 5.33 Mb to *QGpc.spa-3A.2*. The *QGpc.spa-3A.3* peak marker, *IWB39901* (M32), was separated by a physical distance of 59.5 Mb from peak marker *IWB28341* (M31) reported by Fatiukha et al. (2020), therefore *QGpc.spa-3A.3* is likely a novel QTL. Given the close proximity of *QGpc.spa-3A.4* peak marker *IWB58656* (M33) detected in this study of ~7.8 Mb from SNP *IWB35484* (M34) reported to be associated with GPC in durum wheat by Nigro et al. (2019), they are likely the same QTL.

*QGpc.spa-5B* (M36, *IWB10851*) on the short arm of chromosome 5B identified in our study is likely the same QTL as the one reported in the previous study by Rapp et al. (2018) because of their close physical distance. The very close proximity (2.724 kb) of *QGpc.spa-7A* (M49, *IWB4104*) to the SNP (M50, *IWB65659*) associated with GPC (Nigro et al., 2019) indicates these two QTL are the same. Likewise, *QGpc.spa-7A* might be the same QTL as the one (M48, *D1382367*) identified by Rapp et al. (2018) since the distance between the peak markers of these two QTL is 3.0 Mb. In addition, on the long arm of chromosome 7B, *QGpc.spa-7B* (M67, *IWB32614*) is likely the same as the QTL reported by Zhang et al. (2008) and Conti et al. (2011) due to the close proximity of 2.24 Mb and 2.94 Mb to these two reported QTL. A few genes such as Aspartic proteinases involved in amino acid and protein metabolism are in close proximity to *QGpc.spa-2B.1* and *QGpc.spa-3A.3*. Aspartic proteinases have been reported to be involved in proteolytic processing and maturation of storage proteins (Simões and Faro, 2004).

### Conditional QTL

The negative correlation between GY, yield components and GPC in durum wheat and bread wheat is well-documented (DePauw et al., 2007; Suprayogi et al., 2009; Blanco et al., 2012; Bogard et al., 2013). Similarly, in the present study, moderate negative correlations between these two traits were observed in most of the environments. Most of previous studies focused only on the phenotypic correlation of these traits. By comparing traditional and conditional QTL, we tried to elucidate the genetic relationships at individual QTL between these two correlated traits to identify QTL for GPC independent of GY.

When GPC values conditional to GY were used for QTL mapping, eight out of eleven initially mapped QTL for GPC on 1B (*QGpc.spa-1B.1*), 2B (*QGpc.spa-2B.1, QGpc.spa-2B.2, QGpc.spa-2B.3*), 3A (*QGpc.spa-3A.1, QGpc.spa-3A.4*), 5B (*QGpc.spa-5B*), and 7B (*QGpc.spa-7B*) were not detected. This indicates that the expression of these QTL likely depends on GY. Genetic or physiological association might exist between GPC and GY such as a dilution effect of the protein by carbohydrate. A similar observation was reported by Blanco et al. (2012). Some of their initially detected QTL for GPC failed to show significant effects when the GPC values were adjusted against yield components (thousand-kernel weight, grain yield per spike, kernels per spike) then were used for mapping. Such QTL were suggested to represent genes involved in carbohydrate biosynthesis and thus contributing to total grain mass, however, with indirect effect on GPC (Blanco et al., 2012).

The results of the conditional mapping in this study showed a few GPC QTL with no pleiotropic effect on GY, indicating these QTL are independent or partially independent of GY and would have little or no negative effect on GY when selecting for high GPC. The three QTL, *QGpc.spa-3A.2* and *QGpc.spa-3A.3* on chromosome 3A, and *QGpc.spa-7A* on 7A, were detected in conditional mapping but with reduced or slightly reduced effects. This suggested that they function, at least to some degree, independently of GY. The partial independence of the expression of *QGpc.spa-3A.3* and *QGpc.spa-7A* was also reflected by the smaller slope of the regression lines of GPC on GY observed for Hap1 and Hap3 compared with Hap4. It is worth noting that *QGpc.spa-7A* was detected in the same environments by both traditional and conditional mapping with a small reduction in effects, indicating this QTL controls GPC more independently from GY. Both *QGpc.spa-3A.2* and *QGpc.spa-3A.3* were detected only in the environments where no significant (E17 and E18) or weak to moderate (E15 and L15) correlation was observed between GPC and GY. Relatively lower GY was also observed in these environments reflecting the partial dependence on GY for the expression of these GPC QTL. These QTL detected in conditional mapping are of great importance for durum wheat breeding as their incorporation will allow the improvement of GPC without significant compromise on GY.

Five additional QTL for GPC were detected in conditional mapping while they were not detected in traditional mapping. The expression of these QTL may have been masked by GY and was below the detection threshold in traditional GPC mapping; hence, their effects could only be detected with the removal of the confounding effect of GY. A similar observation was reported in a previous study that two additional QTL were detected when GPC values were adjusted to yield components in durum wheat (Blanco et al., 2012). Similarly in bread wheat, three more QTL for GPC were identified by using conditional GPC values on GY and its components (Wang L. et al., 2012).

## Conclusions

The results in this study provide a further understanding of genetic control of GPC in Canadian durum wheat and genetic relationship between GPC and GY. Comparison of the QTL described here with the results previously reported led to the identification of one novel major QTL on 3A (*QGpc.spa-3A.3*) and five novel minor QTL on 1B, 2B and 3A. The conditional and stable QTL (*QGpc.spa-3A.3* and *QGpc.spa-7A*) identified for GPC were partially affected by or independently expressed of GY. These QTL are of great importance and their closely linked markers are useful for MAS for high GPC without concomitant trade-off on GY.

## Data Availability Statement

All datasets generated for this study have been included in the article/Supplementary Material, further inquiries can be directed to the corresponding author/s.

## Author Contributions

YR and RK conceptualized this study. YR, RK, AKS, RD, and RC generated the population and contributed to seed increase of this population. YR, RD, RC, SB, and JS implemented the field trials and phenotyping of the population. WZ, AS, and PF provided the genotyping platform. BY and YR analyzed the data, interpreted results, and contributed to data management and visualization. RK, RD, BXF, and JS contributed to the result interpretation. BY and YR wrote the original manuscript. BY, YR, RK, RD, AKS, BXF, and PF contributed to the review and editing of the manuscript. YR was the principal investigator and supervised the project. All authors contributed to the article and approved the submitted version.

## Conflict of Interest

The authors declare that the research was conducted in the absence of any commercial or financial relationships that could be construed as a potential conflict of interest. The handling editor and reviewer FB declared a past co-authorship with several of the authors RK and AKS.
