# Supplementary material for: Conditional Mapping Identified Quantitative Trait Loci for Grain Protein Concentration Expressing Independently of Grain Yield in Canadian Durum Wheat
Source: Front Plant Sci. 2021 Mar 24;12:642955. doi: 10.3389/fpls.2021.642955 (PMC8024689; doi:10.3389/fpls.2021.642955)
Supplement: Supplementary file 1 [file Data_Sheet_1.PDF]

Conditional Mapping Identified Quantitative Trait Loci for Grain Protein Concentration Expressed Independently of Grain Yield in Canadian Durum Wheat

Yuefeng Ruan[1*†], Bianyun Yu[2*†], Ron E. Knox[1], Wentao Zhang[2], Asheesh K. Singh[1,3], Richard Cuthbert[1], Pierre Fobert[4], Ron DePauw[1,5], Samia Berraies[1], Andrew Sharpe[2,6], Bin Xiao Fu[7], Jatinder Sangha[1]

[1]Swift Current Research and Development Centre, Agriculture and Agri-Food Canada, 1 Airport Road, Swift Current, SK, S9H 3X2, Canada

[2]Aquatic and Crop Resource Development, National Research Council Canada, 110 Gymnasium Place, Saskatoon, SK, S7N 0W9, Canada

[3]Current address: Department of Agronomy, Iowa State University, Ames, IA, USA

[4]Aquatic and Crop Resource Development, National Research Council Canada, 100 Sussex Drive, Ottawa, ON, K1N 5A2, Canada

[5]Current address: Advancing Wheat Technologies, 118 Strathcona Rd, Calgary, AB, T3H 1P3

[6]Current address: The Global Institute for Food Security, 110 Gymnasium Place, Saskatoon, SK, S7N 0W9, Canada

[7]Grain Research Laboratory, Canadian Grain Commission, 303 Main St, Winnipeg, MB, R3C 3G7, Canada

*Corresponding authors:

Bianyun Yu
Bianyun.Yu@nrc-cnrc.gc.ca

Yuefeng Ruan
yuefeng.ruan@canada.ca

[†]These authors have contributed equally to this work.

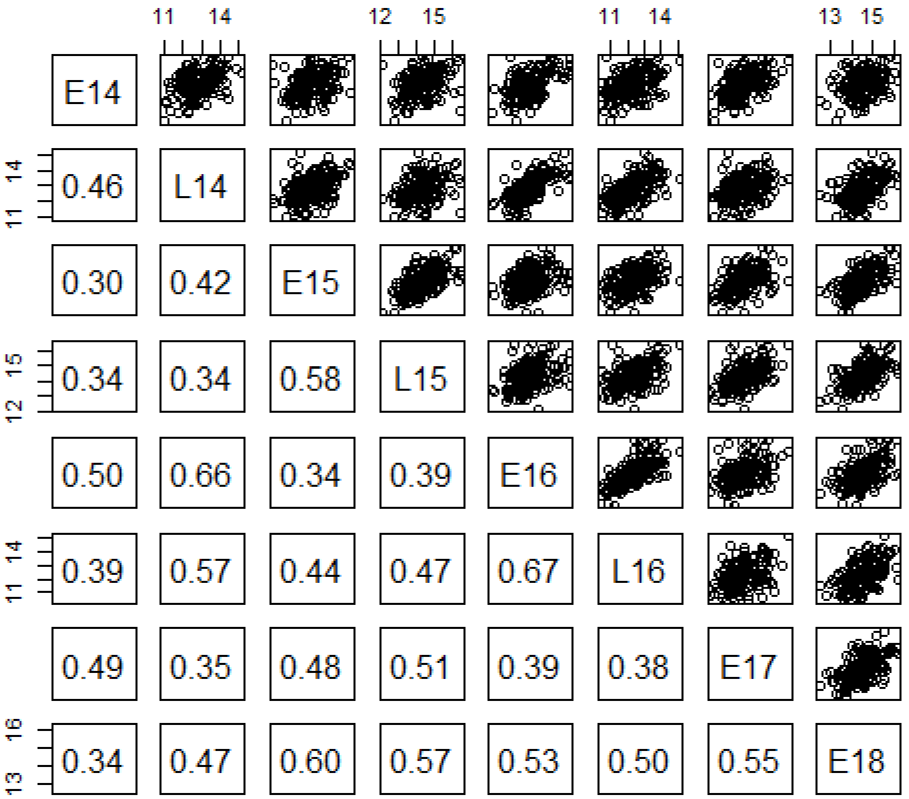

Figure S1 Pearson correlation of grain protein concentration (GPC) of DH lines of the Pelissier × Strongfield population across environments at significance level $p < 0.001$.

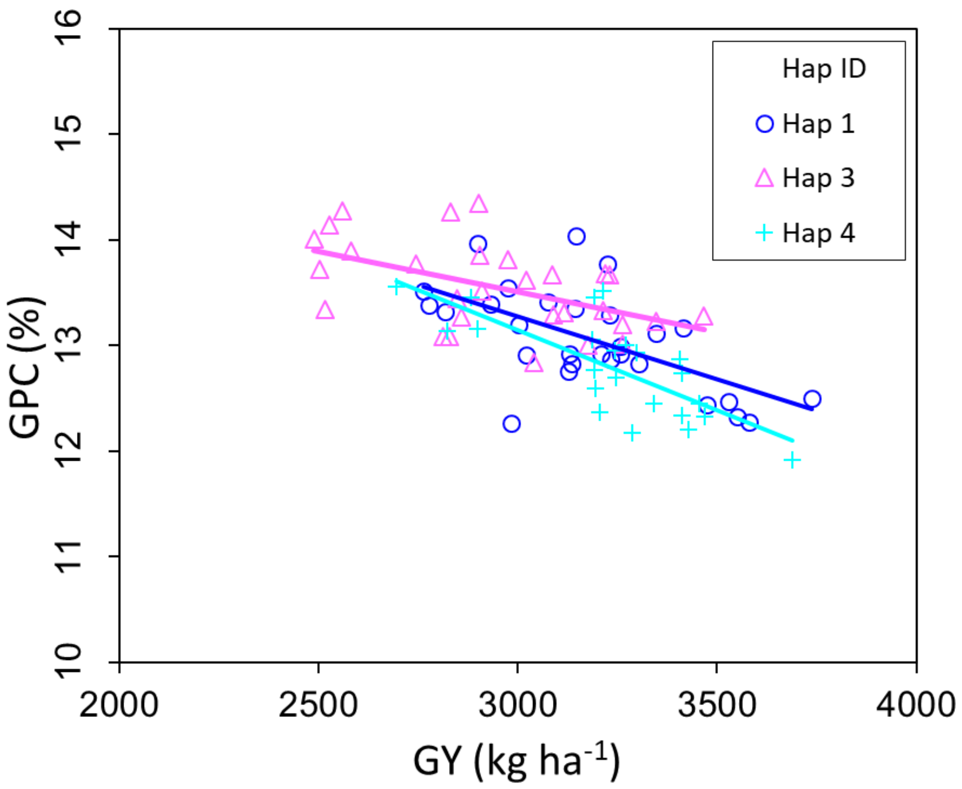

Figure S2 Scatterplot and regression of GPC on GY of haplotype group Hap1 and Hap3, and Hap4. GPC, grain protein concentration; GY, grain yield.

```
                            pid    1 [         .         .         .         .         :         . 60
1 ABI94355.1              100.0%     ---MGSSDSSSGSAPPRHQPPPPQQGSAPELPPGFRFHPTDEELVVHYLKKKAAKVPLPV
2 TRITD2Bv1G082940.1       99.2%     MRSMGSSDSSSGSAPPRHQPPPPQQGSAPELPPGFRFHPTDEELVVHYLKKKAAKVPLPV
3 TRITD2Bv1G082940.2       97.3%     MRSMGSSDSSSGSAPPRHQPPPPQQGSAPELPPGFRFHPTDEELVVHYLKKKAAKVPLPV
4 TRITD2Bv1G082940.3       98.5%     ------------------------------------------------------------
5 TRITD2Bv1G082940.4       95.3%     ----------------------------------------------------------MV

                            pid   61           .         .         .         1         .         . 120
1 ABI94355.1              100.0%     TIITEVDLYKFDPWELPEKATFGEQEWYFFSPRDRKYPNGARPNRAATSGYWKATGTDKP
2 TRITD2Bv1G082940.1       99.2%     TIITEVDLYKFDPWELPEKATFGEQEWYFFSPRDRKYPNGARPNRAATSGYWKATGTDKP
3 TRITD2Bv1G082940.2       97.3%     TIITEVDLYKFDPWELPEKATFGEQEWYFFSPRDRKYPNGARPNRAATSGYWKATGTDKP
4 TRITD2Bv1G082940.3       98.5%     ------------MGAAREKATFGEQEWYFFSPRDRKYPNGARPNRAATSGYWKATGTDKP
5 TRITD2Bv1G082940.4       95.3%     YVCDPVVLVHALARDRSEKATFGEQEWYFFSPRDRKYPNGARPNRAATSGYWKATGTDKP

                            pid  121           .         .         :         .         .         . 180
1 ABI94355.1              100.0%     ILASGCGREKVGVKKALVFYRGKPPKGLKTNWIMHEYRLTDASSSATTSRPPPVTGGSRS
2 TRITD2Bv1G082940.1       99.2%     ILASGCGREKVGVKKALVFYRGKPPKGLKTNWIMHEYRLTDASSSATTSRPPPVTGGSRS
3 TRITD2Bv1G082940.2       97.3%     ILASGCGREKVGVKKALVFYRGKPPKGLKTNWIMHEYRLTDASSSATTSRPPPVTGGSRS
4 TRITD2Bv1G082940.3       98.5%     ILASGCGREKVGVKKALVFYRGKPPKGLKTNWIMHEYRLTDASSSATTSRPPPVTGGSRS
5 TRITD2Bv1G082940.4       95.3%     ILASGCGREKVGVKKALVFYRGKPPKGLKTNWIMHEYRLTDASSSATTSRPPPVTGGSRS

                            pid  181           .         2         .         .         .         . 240
1 ABI94355.1              100.0%     ASL-------RLDDWVLCRIYKKINKAAAGDQQRSMECEDSVEDAVTAYPLYATAGMTGA
2 TRITD2Bv1G082940.1       99.2%     ASL-------RLDDWVLCRIYKKINKAAAGDQQRSMECEDSVEDAVTAYPLYATAGMTGA
3 TRITD2Bv1G082940.2       97.3%     ASLRCTSSSHQLDDWVLCRIYKKINKAAAGDQQRSMECEDSVEDAVTAYPLYATAGMTGA
4 TRITD2Bv1G082940.3       98.5%     ASL-------RLDDWVLCRIYKKINKAAAGDQQRSMECEDSVEDAVTAYPLYATAGMTGA
5 TRITD2Bv1G082940.4       95.3%     ASL-------RLDDWVLCRIYKKINKAAAGDQQRSMECEDSVEDAVTAYPLYATAGMTGA

                            pid  241           :         .         .         .         .         3 300
1 ABI94355.1              100.0%     GAHGSNYDSLLHHQDSHEDNFLDGLLTAEDAGLSAGPTSLSHLAAAARASPAPTKQFLAP
2 TRITD2Bv1G082940.1       99.2%     GAHGSNYDSLLHHQDSHEDNFLDGLLTAEDAGLSAGPTSLSHLAAAARASPAPTKQFLAP
3 TRITD2Bv1G082940.2       97.3%     GAHGSNYDSLLHHQDSHEDNFLDGLLTAEDAGLSAGPTSLSHLAAAARASPAPTKQFLAP
4 TRITD2Bv1G082940.3       98.5%     GAHGSNYDSLLHHQDSHEDNFLDGLLTAEDAGLSAGPTSLSHLAAAARASPAPTKQFLAP
5 TRITD2Bv1G082940.4       95.3%     GAHGSNYDSLLHHQDSHEDNFLDGLLTAEDAGLSAGPTSLSHLAAAARASPAPTKQFLAP

                            pid  301           .         .         .         .         :         . 360
1 ABI94355.1              100.0%     SSSTPFNWLDASTVGILPQARNFPGFNRSRNVGNMSLSSTADMAVDNGGGNAINTMPPFM
2 TRITD2Bv1G082940.1       99.2%     SSSTPFNWLDASTVGILPQARNFPGFNRSRNVGNMSLSSTADMAVDNGGGNAINTMPPFM
3 TRITD2Bv1G082940.2       97.3%     SSSTPFNWLDASTVGILPQARNFPGFNRSRNVGNMSLSSTADMAVDNGGGNAINTMPPFM
4 TRITD2Bv1G082940.3       98.5%     SSSTPFNWLDASTVGILPQARNFPGFNRSRNVGNMSLSSTADMAVDNGGGNAINTMPPFM
5 TRITD2Bv1G082940.4       95.3%     SSSTPFNWLDASTVGILPQARNFPGFNRSRNVGNMSLSSTADMAVDNGGGNAINTMPPFM

                            pid  361           .         .         .         4     ] 406
1 ABI94355.1              100.0%     NHLPMQDGTYHQQHVILGAPLAPEATAAATSAFQHPVQISGVNWNP
2 TRITD2Bv1G082940.1       99.2%     NHLPMQDGTYHQQHVILGAPLAPEATAAATSAFQHPVQISGVNWNP
3 TRITD2Bv1G082940.2       97.3%     NHLPMQDGTYHQQHVILGAPLAPEATAAATSAFQHPVQISGVNWNP
4 TRITD2Bv1G082940.3       98.5%     NHLPMQDGTYHQQHVILGAPLAPEATAAATSAFQHPVQISGVNWNP
5 TRITD2Bv1G082940.4       95.3%     NHLPMQDGTYHQQHVILGAPLAPEATAAATSAFQHPVQISGVNWNP
```

Figure S3 Multiple alignment of protein sequences of various *TRITD2Bv1G082940* transcripts with NAM-B2 from *Triticum turgidum L.* subsp. *durum* (Desf.) Husn. (ABI94355.1). Sequence alignment was conducted using Clustal Omega, https://www.ebi.ac.uk/Tools/msa/clustalo/. Pid, percent of identity.

Supplementary Table S1 Distribution of each quantitative trait loci (QTL) associated marker on the reference genome of durum wheat cv. Svevo

Marker	Marker ID	Marker label	Chr	Start position on Svevo (bp)	QTL source
wPt-3411	wPt-3411	M1	1B	9706246	Suprayogi et al. 2009
wPt-0655	wPt-0655	M2	1B	17438822	Giraldo et al. 2016
BS00110546_51	IWB12562	M3	1B	51853437	In this study
barc18	barc18	M4	1B	87201055	Suprayogi et al. 2009
gwm273, wmc626	gwm273, wmc626	M5	1B	223320855	Conti et al. 2011
IWA141	IWA141	M6	1B	458515820	Nigro et al. 2019
D1112546	D1112546	M7	1B	576212148	Rapp et al. 2018
Tdurum_contig56281_261	IWB72499	M8	1B	605978424	In this study
RAC875_c818_1185	IWB60663	M9	1B	636943315	Fatiukha et al. 2020
IWB41924	IWB41924	M10	1B	655567690	Marcotuli et al. 2017
IWB12436	IWB12436	M11	2B	40767854	Nigro et al. 2019
wmc597	wmc597	M12	2B	69026466	Conti et al. 2011
IWB51809	IWB51809	M13	2B	116757659	Nigro et al. 2019
IWB49035	IWB49035	M14	2B	120769610	Nigro et al. 2019
IWB35350	IWB35350	M15	2B	120779532	Nigro et al. 2019
IWB4951	IWB4951	M16	2B	120779553	Nigro et al. 2019
IWB72906	IWB72906	M17	2B	152341361	Giancaspro et al. 2019
IAAV1903	IWB34469	M18	2B	174375561	In this study
TRITD2Bv1G082940	TRITD2Bv1G082940	2BG082940	2B	218245646	TRITD2Bv1G082940
GPC-B2	GPC-B2	GPC-B2	2B	218245655	Uauy et al. 2006
gwm1249	gwm1249	M19	2B	596754378	Peleg et al. 2009
Ku_c10415_662	IWB38099	M20	2B	624531629	In this study
IWA544	IWA544	M21	2B	647195116	Marcotuli et al. 2017
wmc41	wmc41	M22	2B	695151874	Suprayogi et al. 2009
wmc332	wmc332	M23	2B	706135766	Gadaleta et al. 2011
IWA7955	IWA7955	M24	2B	708554780	Nigro et al. 2019
Xwmc332	Xwmc332	M25	2B	726066228	Blanco et al. 2012
BS00021981_51	IWB6837	M26	3A	56832502	In this study
IWB72484	IWB72484	M27	3A	240526564	Giancaspro et al. 2019
wsnp_Ex_c14681_22747500	IWA1922	M28	3A	488244049	In this study
IWB14495	IWB14495	M29	3A	491373384	Nigro et al. 2019
IWB71028	IWB71028	M30	3A	493578633	Nigro et al. 2019
Excalibur_c6501_477	IWB28341	M31	3A	509910388	Fatiukha et al. 2020
Ku_c70534_1215	IWB39901	M32	3A	569423894	In this study
RAC875_c5056_220	IWB58656	M33	3A	599349622	In this study
IWB35484	IWB35484	M34	3A	607194206	Nigro et al. 2019
D1118885	D1118885	M35	5B	14794967	Rapp et al. 2018
BS00076101_51	IWB10851	M36	5B	17942380	In this study

wmc73	wmc73	M37	5B	80571072	Suprayogi et al. 2009
IWA8604	IWA8604	M38	5B	286325449	Nigro et al. 2019
wg909	wg909	M39	5B	446940179	Gonzalez-Hernandez et al. 2004
gwm499	gwm499	M40	5B	516056119	Conti et al. 2011
IWB61037	IWB61037	M41	5B	607822975	Nigro et al. 2019
IWB72758	IWB72758	M42	5B	608687720	Nigro et al. 2019
IWB6634	IWB6634	M43	5B	618840582	Nigro et al. 2019
wPt-11579	wPt-11579	M44	5B	640182385	Peleg et al. 2009
IWB2716	IWB2716	M45	5B	666780370	Nigro et al. 2019
IWB11571	IWB11571	M46	5B	680547828	Giancaspro et al. 2019
S1279884	S1279884	M47	7A	94792009	Rapp et al. 2018
D1382367	D1382367	M48	7A	108472747	Rapp et al. 2018
BobWhite_c6193_298	IWB4104	M49	7A	111513218	In this study
IWB65659	IWB65659	M50	7A	111515942	Nigro et al. 2019
barc108	barc108	M51	7A	354714037	Suprayogi et al. 2009
IWB20381	IWB20381	M52	7A	673475540	Marcotuli et al. 2017
gwm332	gwm332	M53	7A	681350784	Peleg et al. 2009
D4008953	D4008953	M54	7A	693842061	Rapp et al. 2018
D994221	D994221	M55	7A	704012443	Rapp et al. 2018
D2275833	D2275833	M56	7A	727342153	Rapp et al. 2018
durum_contig10861_942	IWB66787	M57	7B	3883889	Fatiukha et al. 2020
gmw263	gmw263	M58	7B	6861465	Peleg et al. 2009
utv913	utv913	M59	7B	101506378	Blanco et al. 2002
IWB71499	IWB71499	M60	7B	112236693	Marcotuli et al. 2017
IWB71916	IWB71916	M61	7B	441159415	Nigro et al. 2019
Kukri_c14766_484	IWB41262	M62	7B	617849760	Fatiukha et al. 2020
wPt-5922, wPt-9133	wPt-5922, wPt-9133	M63	7B	622210984	Suprayogi et al. 2009
IWB69002	IWB69002	M64	7B	628710157	Giancaspro et al. 2019
Xgwm577-7B	Xgwm577-7B	M65	7B	691657100	Blanco et al. 2006
barc1073-cfa2257	barc1073-cfa2257	M66	7B	701783315	Zhang et al. 2008
GENE-1728_107	IWB32614	M67	7B	704028055	In this study
barc1073, barc340	barc1073, barc340	M68	7B	706972144	Conti et al. 2011
Nitrogen metabolism related SNP markers					
IWB49035			2BS	120769610	Nigro et al. 2019
IWB4951			2BS	120779553	Nigro et al. 2019
IWB35350			2BS	120779532	Nigro et al. 2019
IWB71028			3AL	493578633	Nigro et al. 2019
IWA8604			5B	286325449	Nigro et al. 2019
IWB61037			5BL	607822975	Nigro et al. 2019
IWB72758			5BL	608687720	Nigro et al. 2019
IWA141			1BL	458515820	Nigro et al. 2019
IWA7955			2BL	708554780	Nigro et al. 2019

Chr, chromosome
